# Altered environmental light drives retinal change in the Atlantic Tarpon (*Megalops atlanticus*) over timescales relevant to marine environmental disturbance

**DOI:** 10.1186/s12898-018-0157-0

**Published:** 2018-01-18

**Authors:** Lorian E. Schweikert, Michael S. Grace

**Affiliations:** 1 Department of Biological Sciences, Florida Institute of Technology, 150 W. University Boulevard, Melbourne, FL 32901 USA; 2 Present Address: Department of Biology, Duke University, 130 Science Dr. Durham, Durham, NC 27583 USA

**Keywords:** Vision, Retina, Plasticity, Photoreceptor, Opsin, Spectral sensitivity

## Abstract

**Background:**

For many fish species, retinal function changes between life history stages as part of an encoded developmental program. Retinal change is also known to exhibit plasticity because retinal form and function can be influenced by light exposure over the course of development. Aside from studies of gene expression, it remains largely unknown whether retinal plasticity can provide functional responses to short-term changes in environmental light quality. The aim of this study was to determine whether the structure and function of the fish retina can change in response to altered light intensity and spectrum—not over the course of a developmental regime, but over shorter time periods relevant to marine habitat disturbance.

**Results:**

The effects of light environment on sensitivity of the retina, as well as on cone photoreceptor distribution were examined in the Atlantic tarpon (*Megalops atlanticus*) on 2- and 4-month timescales. In a spectral experiment, juvenile *M. atlanticus* were placed in either ‘red’ or ‘blue’ light conditions (with near identical irradiance), and in an intensity experiment, juveniles were placed in either ‘bright’ or ‘dim’ light conditions (with near identical spectra). Analysis of the retina by electroretinography and anti-opsin immunofluorescence revealed that relative to fish held in the blue condition, those in the red condition exhibited longer-wavelength peak sensitivity and greater abundance of long-wavelength-sensitive (LWS) cone photoreceptors over time. Following pre-test dark adaption of the retina, fish held in the dim light required less irradiance to produce a standard retinal response than fish held in bright light, developing a greater sensitivity to white light over time.

**Conclusions:**

The results show that structure and function of the *M. atlanticus* retina can rapidly adjust to changes in environmental light within a given developmental stage, and that such changes are dependent on light quality and the length of exposure. These findings suggest that the fish retina may be resilient to disturbances in environmental light, using retinal plasticity to compensate for changes in light quality over short timescales.

**Electronic supplementary material:**

The online version of this article (10.1186/s12898-018-0157-0) contains supplementary material, which is available to authorized users.

## Background

The ability of individual organisms to adapt to environmental change (i.e., phenotypic plasticity) supports fitness by allowing the colonization of new environments [1] and survival during environmental disturbance [2]. These unanticipated changes to the physical environment require an organism to either evade environmental stressors or modify its physiology or behavior in order to survive. Aquatic habitats are particularly dynamic because factors including depth [3], suspended sediment [4], and dissolved organic matter can alter underwater light quality [5]. These factors affect light intensity and spectrum, resulting in often predictable and characteristic differences in light quality among aquatic habitats [6]. The dynamic nature of underwater light, along with the critical role of vision in survival may have driven the great diversity and plasticity of retinal form and function observed among fish species [5, 7, 8].

The life history of fish typically includes metamorphosis from a larval stage to one or more juvenile stages before reaching adult sexual maturity. Between these stages, many marine fish migrate to new light environments, which requires changes in visual function [9–16]. Retinal change is thought to occur in anticipation of or in concert with shifts in habitat [17–21], the outcomes of which can be strongly influenced by changes in light exposure over life history [e.g., 22].

Plasticity of retinal development in response to altered environmental light has been observed in a variety of species. Fish reared in atypical lighting conditions through the course of development exhibit differential retinal sensitivity between major life history stages [23–28]. Specifically, differences in rearing light intensity and spectrum affect the spectral sensitivity of photoreceptor cells [27], result in altered retinal architecture [25], and lead to changes in the expression of opsin proteins in the retina [22, 26–28]. However, the capacity of retinal plasticity to compensate for ‘unexpected’ or abrupt changes in underwater light quality within a given life history stage (that is, between major developmental transitions) remains largely unknown.

Major storms, eutrophication events, and other natural and anthropogenic disturbances occur over short timescales and can result in acute and often dramatic shifts in the intensity and spectrum of underwater light [29, 30]. These kinds of disturbances may have profound effects on the abilities of fish to feed, avoid predators, engage conspecifics, and ultimately survive. Several recent studies have shown that retinal gene expression can change in response to abrupt changes in light quality [31–33], in some instances occurring within days or hours of altered light onset [34, 35]. Aside from these studies of gene expression, however, the effects of light change on physiological sensitivity and architecture of the retina remain to be investigated over timescales relevant to environmental disturbance.

The Atlantic tarpon (*Megalops atlanticus*) is a marine fish species that has a long maturation period (∼ 7–10 year; [36]), that includes multiple distinct developmental transitions coupled with movement to new habitats with different light environments. In addition to robust developmental change [15], phenotypic plasticity of the retina would be particularly advantageous to this species because over its long-life span (> 55 years; [37]) *M. atlanticus* occupies and moves between inshore and near-shore waters, both of which are highly susceptible to the light-altering effects of coastal storms, eutrophication, and pollution.

In this study, juvenile *M. atlanticus* were used to investigate how the intensity and spectrum of environmental light affects the fish retina on 2- and 4-month timescales. Electroretinography (ERG) was used to determine the in vivo sensitivity of the *M. atlanticus* retina, allowing for the comparison of retinal function to lighting conditions. Anti-opsin immunofluorescence was used to examine the lengths and distributions of photoreceptors in the *M. atlanticus* retina, in an effort to identify the mechanisms underlying retinal sensitivity shifts. Determining the capacity of the *M. atlanticus* retina for light-induced phenotypic plasticity will indicate the resilience of fish vision to marine disturbances that result in acute changes in environmental light.

## Methods

### Specimen collection and maintenance

The specimens used for each method are summarized in Table 1. Young-of-year juvenile *M. atlanticus* were captured in nursery habitats in Merritt Island, FL by cast net under a Florida Fish and Wildlife (FWC) Special Activity License (SAL-11-1300-SR). Specimens were transported to the Florida Institute of Technology’s (FIT) aquaculture facility and were acclimated to captivity prior to placement in experimental conditions and in vivo testing of the retina. Fish were placed in experimental conditions to test either the effect of light spectra on the retina (i.e., spectral experiment) or the effect of light intensity on the retina (i.e., intensity experiment). In both experiments, fish were housed individually within subdivided 250-gallon, 30 cm-deep, white-walled tanks on a 12-h light, 12-h dark photocycle (LD 12/12), and fed ad libitum. Following experimentation, specimens were humanly euthanized by MS-222 overdose and cervical dislocation prior to enucleation of the eyes. All work reported here was conducted under protocols approved by FIT’s Institutional Animal Care and Use Committee (Animal Welfare Assurance #141016).

**Table 1:** Specimens of *Megalops atlanticus* used in this study

Experiment	*N*	Light condition	TL (cm)
Spectral^a,b^	16	Red	19.4, 17.9, 18.4 19.8, 18.6, 19.2, 20.9
		Blue	21.4, 17.9, 17.5, 18.4 20.7, 16.3, 16.5, 19.7
Intensity^a,b^	8	Bright	19.4, 22.0, 24.0, 24.4
		Dim	23.3, 24.2, 22.9, 23.6

*TL* total length of specimens, sample sizes and experimental conditions are provided

^a^Specimens used in electroretinography

^b^Specimens used in immunofluorescence

### Experimental design

**Fig. 1:**
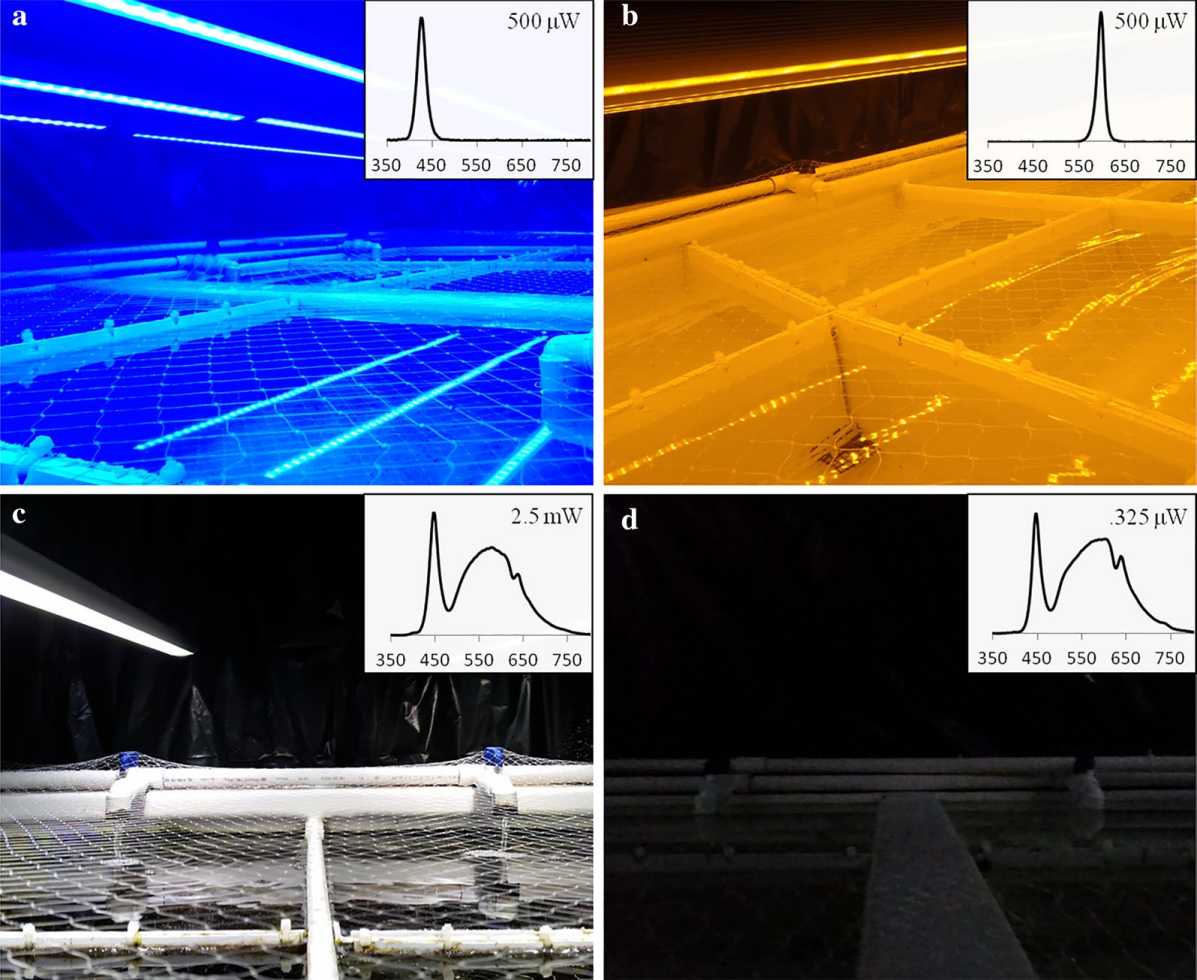
Lighting conditions of spectral and intensity experiments. In the spectral experiment, juvenile *Megalops atlanticus* were kept in lighting conditions of different spectra but identical irradiance (500 µW s^−1^ cm^−2^). The ‘blue’ condition (**a**) and ‘red’ condition (**b**) were centered on 420 nm light and 590 nm light, respectively. In the intensity experiment, *M. atlanticus* were kept in lighting conditions of different irradiances but identical spectra. The ‘bright’ condition (**c**) and ‘dim’ condition (**d**) consisted of 2.5 mW s^−1^ cm^−2^ and .325 µW s^−1^ cm^−2^ white light, respectively. Insets: emission spectra and irradiance values for each condition, y-axes are normalized irradiance (μW s^−1^ cm^−2^)

For the spectral experiment, 16 *M. atlanticus* were placed in conditions of different light spectra, but near identical irradiance (Fig. 1a, b). Light intensity in each condition was 500 μW s^−1^ cm^−2^ at the water’s surface, provided by sixteen 1.3 m-long, custom-made LED light bars (BML Horticulture, Austin, TX, USA). ‘Red’ and ‘blue’ conditions consisted of 50 nm bandwidth spectra centered on 590 and 420 nm, respectively (Fig. 1). These spectra are detectable on the extreme ends of juvenile *M. atlanticus* wavelength sensitivity based upon previous microspectrophotometric (MSP) analyses [15]. Thus, these light conditions should activate long-wavelength sensitive (LWS) and short-wavelength sensitive (SWS) cone types independently. After 60 and 120 days, the spectral sensitivity of four *M. atlanticus* from each condition was tested in vivo by electroretinography, after which the fish were euthanized, eyecups removed, and their eyes fixed for histological analyses. Of the sixteen fish in the spectral experiment, one died from natural causes during the course of the experiment, so a total of three fish (as opposed to four) were tested as part of the red condition at the first timepoint.

For the intensity experiment, eight *M. atlanticus* were placed in conditions of different light irradiance, but near-identical spectra (Fig. 1c, d). The light spectrum of each condition was engineered to approximate that of natural sunlight, and were provided by four 1.3 m-long, custom-made ‘Solar-Max’ LED light bars (BML Horticulture Inc., Austin, TX, USA). Preliminary analysis of *M. atlanticus* retinal sensitivity by electroretinography included differentiation of light activation of rod- and cone-based systems by analysis of a-wave and b-wave amplitudes [38, 39]. An abrupt change occured at ~ 3 μW s^−1^ cm^−2^ (Additional file 1: Figure S1). Thus, light intensities for the ‘bright’ and ‘dim’ light conditions were set at 2.5 mW and .325 μW s^−1^ cm^−2^, respectively, in order to activate cone-based and rod-based vision. Light intensities were measured and set using a radiant power meter with a silicon photodiode (Ophir Photonics, North Andover, MA). Changing the intensity of light bar output did not affect the light spectrum (measured with a Hyper OCR spectroradiometer, Satlantic LP, Halifax, Nova Scotia) between the two lighting conditions. After 60 days, the retinal sensitivity of the four *M. atlanticus* from each condition was tested in vivo by electroretinography, after which the fish were euthanized, eyes were removed, and eyecups prepared and fixed for immunofluorescence analyses (see below).

### Electroretinography

For fish in the spectral experiment, electroretinography (ERG) was used to determine spectral sensitivity of the retina. Fish were anesthetized with 75 mg L^−1^ tricaine methanesulfonate (MS-222) in seawater, transferred to a flow-through apparatus for perfusion of the gills, and dark-adapted for 45 min. A silver-wire recording electrode was placed on the cornea of one eye, a reference electrode was placed just posterior to that eye, and a ground electrode was placed in seawater adjacent to the body. Signals were amplified using an AM Systems (Carlsborg, WA, USA) differential microelectrode amplifier (gain: 1000x; bandpass 1 Hz-1 kHz), and digitized by an iWorx (Dover, NH, USA) amplifier before recording on a laptop using iWorx Labscribe Software. Stimulus light from a QTH lamp was passed through a grating monochromator, an electronic fast-acting shutter (Uniblitz, Rochester, NY) and a 60 cm liquid light guide (Oriel Instruments, Irvine, CA, USA). Light intensity was measured using a radiant power meter (Ophir Photonics, North Andover, MA) and adjusted manually with a slide bar aperture.

The fish retina was stimulated with 100 ms flashes of monochromatic light at increasing irradiances until a criterion retinal response (~ 30 μV) was attained at each wavelength from 350 to 650 nm in 50 nm increments. A criterion response curve was generated by recording the irradiance (in μW s^−1^ cm^−2^) necessary to elicit the criterion response for each wavelength increment. After conversion to photon flux, the inverses of these values were normalized and plotted to generate spectral sensitivity curves. Statistical comparison of the peak wavelength sensitivity (λ_max_, nm) of fish from each lighting condition was performed using a 2 × 2 multifactorial ANOVA to test the effects of spectrum and time on spectral sensitivity. Assumptions (homogeneity of variances and normality of the residuals) were met for this test and all other multifactor ANOVA tests.

For fish in the intensity experiment, white-light sensitivity of the retina was tested by ERG. Following 45 min of dark adaption, fish retinas were stimulated with 100 ms flashes of white light at increasing irradiances until a criterion response (μV) was achieved. After each stimulus, the difference in voltage from the trough of the ERG a-wave to the peak of the b-wave was measured, and irradiance was increased until a criterion response 80 μV above baseline noise was attained. The irradiance (in μW s^−1^ cm^−2^) required to elicit the criterion response was converted to photons s^−1^ cm^−2^. For fish in each condition, the required irradiance was averaged and compared statistically. Following a natural log transformation of the data, an Independent Samples T-test (for which all assumptions of the data were met) was used to test the effect of light intensity on the white-light sensitivity of the retina.

### Immunofluorescence

The effects of light spectra on photoreceptor outer segment length and distribution were determined by immunofluorescence. Eyecups were fixed in a solution of 4% paraformaldehyde and 15% picric acid in .1 M sodium phosphate buffer. Following a minimum of 48 h of fixation, eyecups were infiltrated with 25% sucrose in .1 M Tris buffer, then embedded in Tissue-Tek. Frozen 20 μm-thick cross-sections were cut in the dorso-ventral plane on a CM1850 cryostat (Leica Biosystems, Buffalo Grove, IL, USA), thaw-mounted onto gelatin–coated glass microscope slides, and dried at room temperature overnight. Slides were then placed into fixative for 1 h, followed by four, 15 min washes in Tris-buffered saline (.5 M Trizma buffer, .9% NaCl, pH 7.4). Primary antisera were diluted in Tris-buffered saline (TBS) containing .25% λ-carrageenan, 1% bovine serum albumin, and .3% Triton X-100, and applied to the slides for overnight incubation (minimum 8 h) at room temperature. After four, 15 min rinses in TBS, slides were incubated for 1 h at room temperature in fluorophore-conjugated secondary antisera. Controls for each specimen included omission of the primary antisera, and single-labeling with each primary and secondary combination. Following four rinses in Tris-buffered saline, slides were coverslipped with Slowfade Gold mounting medium with DAPI nucleic acid label (Life Technologies, Grand Island, NY) and imaged using EZ-C1 software on a Nikon C1Si upright confocal laser-scanning microscope (Nikon Instruments, Melville, NY).

Retinas were double-labeled with well-characterized antisera against rod and cone opsins [e.g., 16, 40]. Photoreceptor subtypes were labeled using a mouse monoclonal anti-rhodopsin antibody (MAB5316, 1:500 dilution, RRID AB_2156055, EMD Millipore, Billerica, MA), and a rabbit polyclonal anti-LWS cone opsin antiserum (CERN906 1:1000 dilution, RRID inapplicable, donated by W.J. DeGrip, University of Nijmegen, Nijmegen, Netherlands). Primary antisera were labeled with secondary antisera that were conjugated to Alexa Fluor fluorescent dyes (goat anti-rabbit IgG conjugated to Alexa-488, RRID AB_143165, and goat anti-mouse IgG conjugated to Alexa-555, RRID AB_141780; Life Technologies, Grand Island, NY).

For the retina of fish from the spectral experiment, cone photoreceptor abundances (number per 50 μm linear expanse of retinal section) were counted using NIS Elements (Nikon). Cone photoreceptor abundances in the dorsal and ventral aspects of the *M. atlanticus* retina were compared using a Mann–Whitney U Test. Cone photoreceptor abundances and outer segment lengths were compared using a 2 × 2 multifactorial ANOVA to test the effects of spectrum and time on photoreceptor distribution. Because the *M. atlanticus* retina only contains ‘single’ cone photoreceptors throughout life [15], counting photoreceptor outer segments was an appropriate measure of photoreceptor cell abundance. For the retina of fish from the intensity experiment, photoreceptor abundances were counted considering three different metrics. In juvenile *M. atlanticus*, the rod photoreceptors are approximately half the length of cone photoreceptors and stacked into morphological elements known as rod bundles [15]. Therefore, the number of cones per 50 μm linear expanse of retinal section, rod bundles per 50 μm of retinal section, and cones per of rod bundle were determined using NIS Elements (Nikon). These three measures of the *M. atlanticus* retina, as well as cone outer segment lengths were then compared between fish in bright and dim light conditions using three separate Independent Samples T-tests, for which all assumptions of the data were met.

## Results

### Spectral experiment

**Fig. 2:**
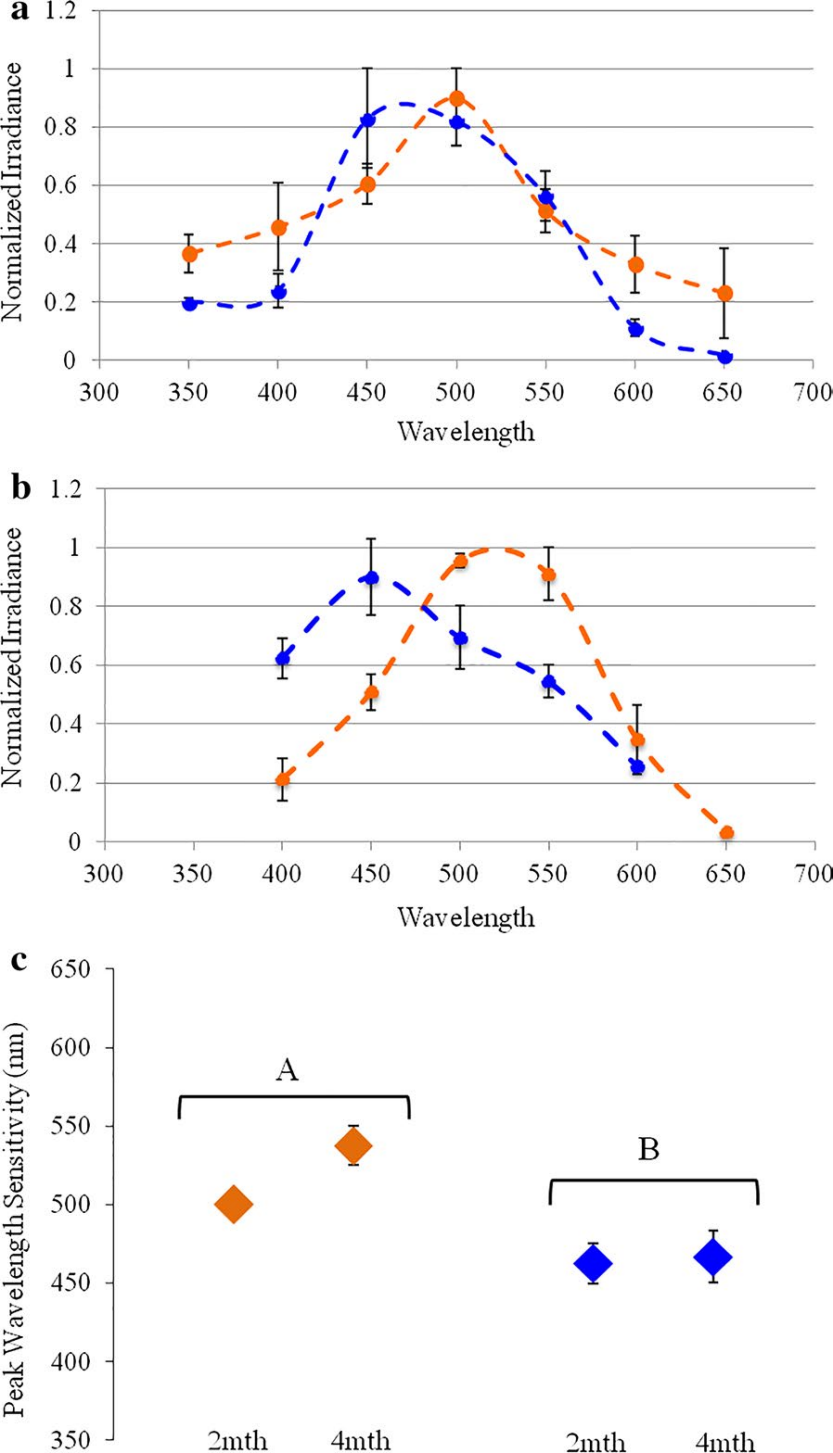
Effects of light spectrum and exposure time on the spectral sensitivity of juvenile *Megalops atlanticus*. Spectral sensitivity curves of *M. atlanticus* from ‘red’ and ‘blue’ light conditions were determined by electroretinography (ERG) and plotted at 2 month (**a**) and 4 month (**b**) time points. In **a**, **b** the average amount of light required to produce a criterion response of the retina was determined in 50 nm increments across the visible light spectrum. The average peak wavelength of sensitivity is shown for fish in each condition (**c**). The effects of light color and exposure time on peak spectral sensitivity of the retina was compared by a 2 × 2 multifactor ANOVA. Brackets with lettering indicate significant difference in sensitivity between spectral conditions. Error bars indicate standard error of the mean

The mean peak wavelength sensitivity (λ_max_) of *M. atlanticus* held in the ‘red’ condition became significantly longer in wavelength than the mean λ_max_ of *M. atlanticus* held in the ‘blue’ condition (*F* = 18.57, *p* = .002; Fig. 2). After 2 months, the mean λ_max_ of fish in the red condition was 500 nm (s.e.m. ± 0 nm), while the mean λ_max_ of fish in the blue condition was 463 nm (s.e.m. ± 12.5 nm). After 4 months, the mean λ_max_ of fish in the red condition increased to 538 nm (s.e.m. ± 12.5 nm), while the mean λ_max_ of in the blue condition remained unchanged at 467 nm (s.e.m. ± 16.7 nm). Retinal criterion response values were never reached for the 350 and 650 nm stimuli for fish from the blue condition and for 350 nm stimulus for fish in the red condition at 4 months due to low retinal sensitivity at those values. There was no significant difference in λ_max_ between 2 and 4 months within each spectral condition and no significant interaction of light spectrum and time was detected.

**Fig. 3:**
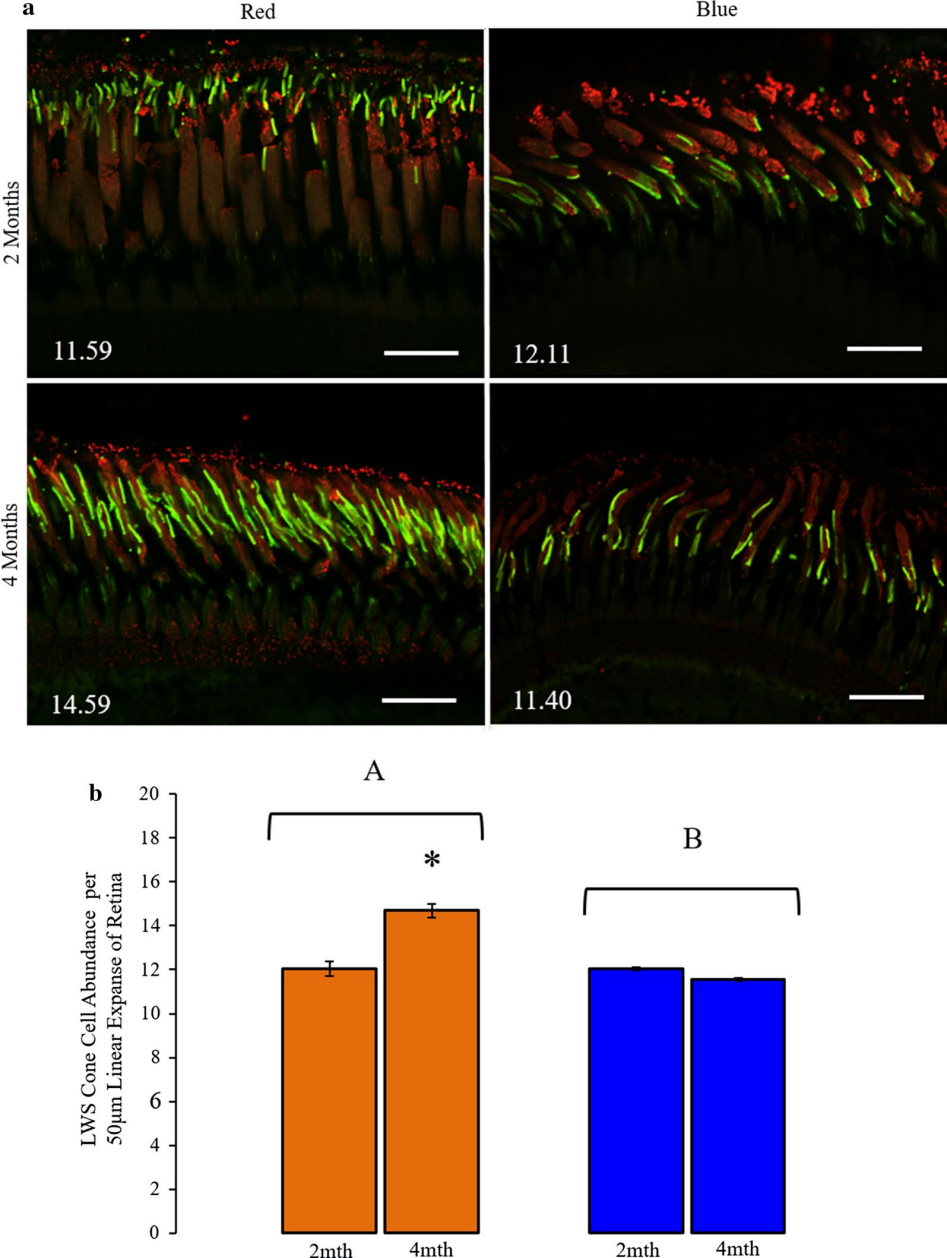
Effects of light spectrum and exposure time on the proportion of cone photoreceptor sensitivity types in juvenile *Megalops atlanticus*. **a** Green fluorescence indicates long-wavelength sensitive (LWS) cone opsin immunoreactivity; red fluorescence indicates rod opsin immunoreactivity. Inset values indicate the average of putative LWS cone photoreceptor abundances per 50 μm linear expanse of retina. Scale bar indicates 50 μm. **b** The effects of light color and exposure time on LWS cone photoreceptor abundance per 50 μm expanse of retina was compared by a 2 × 2 multifactor ANOVA. Brackets with lettering indicate significant difference in sensitivity between spectral conditions. Asterisk indicates significant difference between time points. Error bars indicate standard error of the mean

Strong anti-rhodopsin and anti-cone opsin immunofluorescence was observed in the *M. atlanticus* retina, revealing appropriate rod- and cone-specific photoreceptor outer segment morphologies (Fig. 3). Cone outer segment lengths did not differ between spectral condition or over time; averaging 18.9 µm (s.e.m. ± .35) and 19.1 µm (s.e.m. ± .26) for fish in the red and blue conditions, respectively. In addition, cone photoreceptor abundance did not differ between the dorsal and ventral region of the retina. Comparing cone photoreceptor abundance between spectral conditions, however, indicated that LWS cone abundance was higher for fish in the red condition than for fish in the blue condition (*F* = 53.0, *p* < .001) and that cone photoreceptor abundance differed across time (*F* = 25.24, p < .001). The highest abundance of cone cells was found in fish at 4 months in the red condition (14.68 cells/50 μm; s.e.m. ± .30), and the lowest abundance of cells was found in fish at 4 months in the blue condition. Additionally, an interaction of the effect of light spectrum and time on cone abundance was detected (*F* = 52.04, *p* < .001).

### Intensity Experiment

**Fig. 4:**
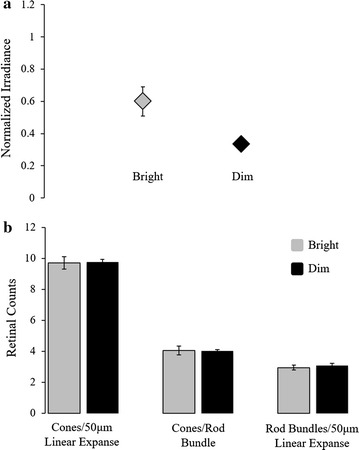
Effects of light irradiance on retinal white-light sensitivity and rod and cone photoreceptor abundance. **a** White-light sensitivity of juvenile *Megalops atlanticus* maintained in ‘bright’ and ‘dim’ light conditions and tested by electroretinography (ERG). Average irradiances (µW s^−1^ cm^−2^) required to elicit an 80 µV retinal response from fish held under each condition were significantly different between ambient illumination levels. Error bar obscured by the marker for fish in the ‘dim’ condition. **b** Relative rod and cone photoreceptor abundance in the retinas of fish held under ‘bright’ and ‘dim light conditions. Error bars indicate standard error of the mean

The electroretinograms of *M. atlanticus* in the intensity experiment indicated a difference in the white-light sensitivity of fish held in the ‘bright’ and ‘dim’ light conditions (Fig. 4a). Following 45 min of dark adaption, fish that had been held in the bright condition required an average of .6 μW s^−1^ cm^−2^ (s.e.m. ± .09 μW) of white light to elicit an 80 μV response of the retina, while fish from the dim condition required an average of only .3 μW s^−1^ cm^−2^ (s.e.m. ± .03 μW) of white light to elicit the same response. This difference in the irradiance required to elicit the criterion response between the two conditions was significant (*t* = 2.573, *p* = .040).

Comparing cone photoreceptor abundance after the intensity experiment indicated that cone abundance per 50 μm linear expanse of retina did not significantly differ between fish in the bright and dim conditions (Fig. 4b); 9.7 cells/50 μm (s.e.m. ± .80 cells) and 9.8 cells/50 μm (s.e.m. ± .32 cells) for fish in the bright and dim conditions, respectively. Congruently, cone abundance per rod bundle and rod bundle abundance (per 50 μm linear expanse of retina) also did not differ significantly between the two conditions; 4.1 cells/50 μm (s.e.m. ± .56 cells) and 4.0 cells/50 μm (s.e.m. ± .20 cells) for fish in the bright and dim conditions, respectively. Rod bundle abundance per 50 μm linear expanse of retina did not significantly differ between fish in the bright and dim conditions: 2.9 bundles/50 μm (s.e.m. ± .33) and 3.1 bundles/50 μm (s.e.m. ± .30), respectively. Cone photoreceptor outer segment lengths did not differ between the two conditions, averaging 18.3 µm (s.e.m. ± .4) and 18.4 (s.e.m. ± .49) for fish in the bright and dim light conditions, respectively.

## Discussion

The goal of this study was to determine the capacity of the marine fish retina to change over short (i.e., 2–4 month) timescales in response to changes in the intensity and spectrum of environmental light. Over the full time course of fish development, retinal function is known to change between life history stages [e.g., 11–13, 15, 16, 21], the outcomes of which can be influenced by light exposure over time [23–28, 41]. Aside from studies of gene expression (e.g., [31]), it remained unclear if disturbances to environmental light quality could drive changes in retinal form and function *between* major developmental transitions (i.e., within a particular developmental stage, and particularly over very short timescales relative to the full course of development). The results of the present study indicate that photoreceptor abundance, absolute sensitivity and spectral sensitivity of the *M. atlanticus* retina all undergo significant change in response to light condition within a 2- to 4-month timescale during the juvenile developmental stage. These changes depend upon the intensity and spectrum of lighting conditions and length of exposure.

### Retinal response to light conditions

Spectral condition was found to drive changes in the peak wavelength sensitivity (λ_max_) of the *M. atlanticus* retina. Over time, fish in the ‘red’ condition became more sensitive to long-wavelength light than fish in the ‘blue’ condition. This sensitivity difference between the fish in the two color conditions was likely driven by cone photoreceptor class redistribution in the retina, as fish from the red condition were found to have a significantly greater abundance of LWS cones relative to fish held in the blue condition. Spectral conditions also may have altered SWS cone photoreceptor abundances over time, but none of the SWS cone opsin antisera tested labeled the *M. atlanticus* retina.

It remains unknown whether the observed changes in LWS cone photoreceptor abundance were due to expression of new opsins within existing photoreceptor cells or neurogenesis of new cells altogether. The retinas of teleost fish grow continuously through persistant neurogenesis throughout life [42]. During this growth, cones form at the retinal boundary, the circumferential germinal zone, while rods can form there, as well as from precursor cells throughout the retina [7, 43]. Whether the retinal changes seen in this and in other studies of retinal plasticity are due to cone cell neurogenesis in the central retina remains to be elucidated.

Exposure of *M. atlanticus* to different irradiance levels caused absolute (white-light) sensitivity of the retina to change substantially. Fish held in the bright light condition required twice the irradiance to produce the same retinal response as fish held in the dim light condition. Yet rod and cone photoreceptor abundance, measured per unit retinal expanse and relative to each other, did not change in response to lighting condition. The observed sensitivity difference may have been a product of a variety of cellular and biochemical mechanisms including modulation of the regeneration rate of light-sensitive visual pigment molecules in photoreceptors [e.g., 44, 45], repositioning of rod and cone outer segments, movement of melanin pigment granules in the retinal pigmented epithelium (in many non-mammalian vertebrates; [e.g., 46]), chemical (de)sensitization of the retina [e.g., 47, 48], or rewiring of the inner retina.

### Plasticity of the marine fish retina

Multiple previous studies of fish retinal phenotypic plasticity demonstrate retinal change in response to altered light spectra that span the entirety of development or key developmental transitions. These studies reported structural and functional changes including the relative proportions of photoreceptor sensitivities determined by microspectrophotometry (MSP; [27]), relative expression of opsin genes [22, 26], behavioral measures of color sensitivity [25], photoreceptor morphology, and even synaptic architecture of the inner retina [24]. More recently, studies of retinal plasticity have demonstrated changes in retinal gene expression in response to short-term changes in environmental light [31–33]. The results presented here expand upon those studies, showing that in vivo retinal sensitivity and photoreceptor distribution can change in response to acute changes in environmental light that occur within a given developmental stage, between major developmental transitions. Significant retinal change occurred in only 2–4 months within the full developmental regime of *M. atlanticus* that encompasses 7–10 years [36]. These results suggest that retinal plasticity may support rapid retinal change and perhaps improved vision for survival in the face of natural and anthropogenic aquatic habitat disturbances that alter intensity and spectrum of underwater light.

## Conclusions

Elucidating the capacity of the fish retina to change in response to altered lighting conditions is important for our understanding of fish resilience to disturbances in the marine environment. Natural and anthropogenic disturbances to marine habitats (e.g., algal blooms caused by eutrophication and storm-induced turbidity changes) can lead to significant and rapid changes in the intensity and spectrum of underwater light [29, 30]. The attenuation of light and chromatic changes associated with marine disturbances may place significant pressure on the visual systems of fish, and thus survival may depend at least in part upon the ability of fish visual systems to change rapidly [49]. The results presented here indicate plasticity of the fish retina in response to acute changes in environmental light over a few months, only ~ 2% of the time it takes *M. atlanticus* to fully develop. Through a combination of changes in photoreceptor distribution and modification of retinal sensitivity, the *M. atlanticus* retina may be able to compensate for new lighting conditions on a timescale relevant to the ecological impact of habitat disturbance. The relatively short-term changes in sensitivity described here may allow *M. atlanticus* to remain efficient predators, avoid potential predators, and interact productively with conspecifics even in the face of significant, rapid environmental change.

## Additional file

**Additional file 1: Figure S1.** Electroretinographic data indicating light intensities required to activate the rod- and cone-based retinal responses in *M. atlanticus*. Individual ERG traces in response to white light irradiances from .5 to 3 µW s^−1^ cm^−2^ following 45 min of dark-adaption (A−D). Flash intensities up to 2 μW s^−1^ cm^−2^ light stimuli produced putative rod responses. Evocation of a defined a-wave (black arrow) and a greater amplitude and temporally broader b-wave (white arrow) indicates activation of cone responses [38, 39]. Bottom: Irradiance levels chosen for ‘dim’ and ‘bright’ experimental conditions (dotted lines; .325 µW s^−1^ cm^−2^ and 2.5 mW s^−1^ cm^−2^, respectively).
