# Supplementary figures and images for: Altered environmental light drives retinal change in the Atlantic Tarpon (Megalops atlanticus) over timescales relevant to marine environmental disturbance
Source: BMC Ecol. 2018 Jan 18;18:1. doi: 10.1186/s12898-018-0157-0 (PMC5774114; doi:10.1186/s12898-018-0157-0)

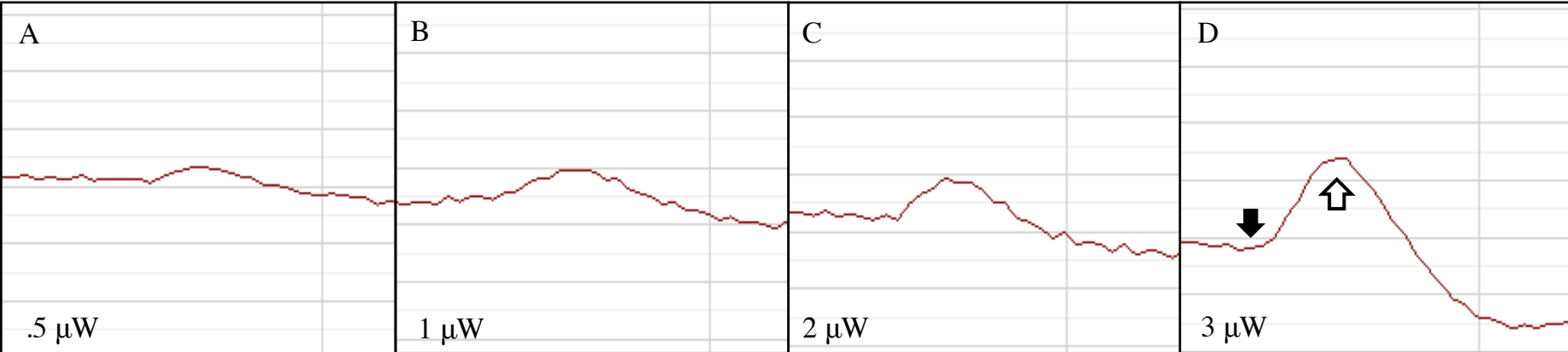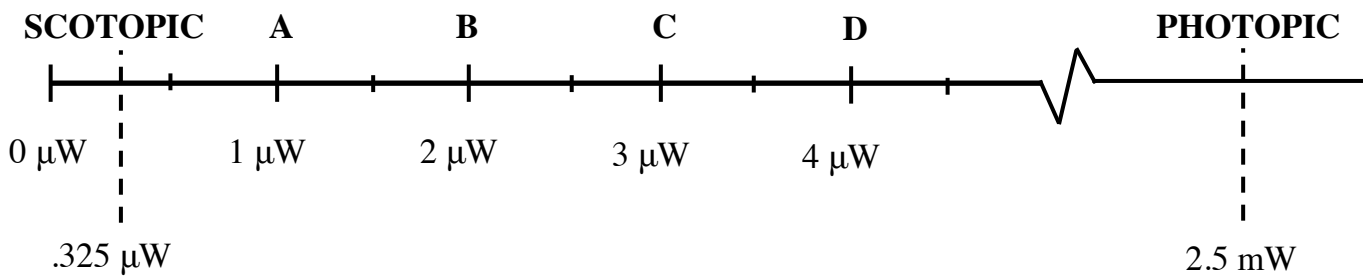

Supplement: Supplementary file 1 — Additional file 1: Figure S1. Electroretinographic data indicating light intensities required to activate the rod- and cone-based retinal responses in M. atlanticus. Individual ERG traces in response to white light irradiances from .5 to 3 µW s−1 cm−2 following 45 min of dark-adaption (A−D). Flash intensities up to 2 μW s−1 cm−2 light stimuli produced putative rod responses. Evocation of a defined a-wave (black arrow) and a greater amplitude and temporally broader b-wave (white arrow) indicates activation of cone responses [38, 39]. Bottom: Irradiance levels chosen for ‘dim’ and ‘bright’ experimental conditions (dotted lines; .325 µW s−1 cm−2 and 2.5 mW s−1 cm−2, respectively). [file 12898_2018_157_MOESM1_ESM.pdf]
